# From Data to Discovery: Characterizing the Scope and Limitations of Seedling Trait Studies in the U.S. With the EPiC Database

**DOI:** 10.1002/ece3.74132

**Published:** 2026-09-14

**Authors:** Nia M. Johnson, Leah J. Lenzo, Amelia G. Renner, Sofia M. A. Al‐Shayeb, Julie E. Larson, Magda Garbowski, Mandy L. Slate, Agneray C. Alison, Sarah C. Barga, Tayah C. Carlisle, Andrew Currens, McKenzie Dorrell, Caroline A. Havrilla, Marina L. LaForgia, Amanda Paradis, Moira M. Newman, Samantha Rosa, Michaela Sershen, Daniel E. Winkler, Halo Wood, Alicia J. Foxx

**Affiliations:** ^1^ The Nature Conservancy Arlington Virginia USA; ^2^ Chicago Botanic Garden Negaunee Institute for Plant Conservation Science and Action Glencoe Illinois USA; ^3^ University of Nevada Reno Nevada USA; ^4^ Department of Biological Sciences University of Illinois at Chicago Chicago Illinois USA; ^5^ University of Washington, School of Environmental and Forest Sciences Seattle Washington USA; ^6^ Department of Animal and Range Sciences New Mexico State University Las Cruces New Mexico USA; ^7^ Department of Evolution, Ecology, and Organismal Biology Ohio State University Columbus Ohio USA; ^8^ University of Nevada, Reno, Biology Reno Nevada USA; ^9^ USDA Forest Service, Rocky Mountain Research Station Cedar City Utah USA; ^10^ Northwestern University, Plant Biology and Conservation Evanston Illinois USA; ^11^ Department of Forest and Rangeland Stewardship Colorado State University Fort Collins Colorado USA; ^12^ Sacramento State University, Biological Sciences Sacramento California USA; ^13^ Amherst College, Biology Amherst Massachusetts USA; ^14^ Department of Biological Sciences, Biology‐Psychology Building University of Maryland College Park Massachusetts USA; ^15^ U.S. Geological Survey, Southwest Biological Science Center Tucson Arizona USA

**Keywords:** early life history, functional traits, restoration, seedling, synthesis

## Abstract

The seedling stage is a uniquely vulnerable and influential period of the plant life cycle with outsized impacts on plant recruitment, and population and community dynamics. Despite this, plant morphology, structure, and function during the seedling stage often differ markedly from later developmental stages, complicating generalizations about ecological strategy across ontogeny. Trait‐based ecology offers a valuable framework to understand how functional variation shapes plant performance at early‐life stages and its ecological consequences. Yet, trait‐based approaches applied at the seedling stage remain limited and inconsistent across studies. To address these limitations, our systematic review of 181 published studies investigated seedling traits of native grass, forb, and shrub species across diverse environmental conditions in the continental United States. Following extraction of covariates, we identified emergent patterns, limitations, and opportunities to expand seedling functional ecology, particularly in relation to regeneration strategies. We found that most studies focused on individuals younger than 90 days old, with only 24% of studies measuring seedling traits beyond this window. Most research emphasized morphological traits (36%), followed by performance/growth (34%), physiological (15%), and phenological traits (13%), with aboveground biomass as the most studied trait. We identified nearly 300 traits but suggest future research efforts focus on key traits for better comparability of seedling responses. Most taxa were in Poaceae (24%) and Asteraceae (25%) families, and nearly 60% of field studies were conducted in dryland ecoregions. Water availability was the most frequently manipulated abiotic factor (39%), while physical disturbance—despite its central role in ecosystem dynamics—was rarely examined (5%). To advance seedling functional ecology and its relevance in applied and community assembly contexts, greater representation of phenological and belowground traits is needed alongside evaluations in diverse environments in the US. Measuring traits that span distinct ontogenetic stages, ecoregions, and taxonomic groups will advance understanding of plant regeneration under accelerating change.

## Graphical abstract

Our systematic review of 181 published studies investigated seedling traits of native grass, forb, and shrub species across diverse environmental conditions in the continental United States. We found that most studies focused on individuals younger than 90 days old, with only 24% of studies measuring seedling traits beyond this window. Most research emphasized morphological traits (36%), followed by performance/growth (34%), physiological (15%), and phenological traits (13%), with aboveground biomass as the most studied trait. More work is needed to measure traits that span distinct ontogenetic stages; ecoregions and taxonomic groups will advance understanding of plant regeneration under accelerating change.

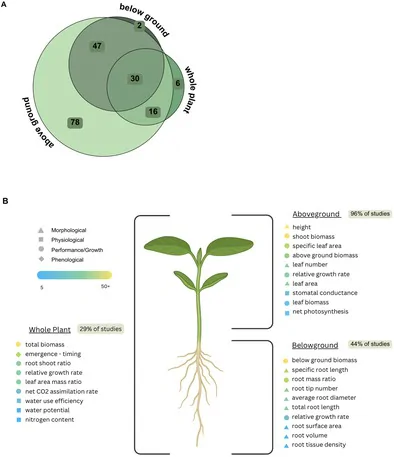

## 1 Introduction

Advanced understanding of plant recruitment is central to mitigating biodiversity loss and maintaining ecosystem functions and services under intensifying land degradation and global change (Miller et al. 2017; Gann et al. 2019; Di Sacco et al. 2021). Seedlings are central to vegetation recovery after disturbance and degradation, as recruitment represents the foundational process for reassembling plant communities (e.g., Grubb 1977; Eriksson and Ehrlén 2008). However, the rarity of successful recruitment—especially in harsh and variable environments—often hinders natural regeneration and active restoration efforts (Merritt and Dixon 2011; James et al. 2011; Shackelford et al. 2021). The functional traits of seedlings—spanning physiological, morphological, phenological, and growth‐related aspects of plant biology—may mediate their responses to environmental stressors (Grossnickle 2012; Larson et al. 2015; Funk et al. 2017), yet a paucity of data for seedlings limits the ability of ecologists to quickly and accurately apply understanding of trait‐based recruitment to challenges associated with land management and restoration.

The work to leverage seedling traits to improve restoration outcomes could be bolstered if seedlings are ecologically matched to their environments (Johnson et al. 2010; Balazs et al. 2020; Shackelford et al. 2021)—and we focus on research conducted in the United States to highlight how and the need of seedling traits being shown to influence plant performance in species used in restoration (e.g., Leger et al. 2019; Foxx and Kramer 2020). However, trait evaluation in early plant life stages has historically lagged behind established, adult stages (Larson and Funk 2016a; Saatkamp et al. 2019). Compiling information on existing seedling trait studies allows for a timely assessment of patterns in the prevalence of different traits and environments being represented and prioritized thus far. A phylogenetic perspective on this compilation can provide additional insight into whether seedling trait knowledge is beginning to span diverse lineages or is concentrated in certain species or taxonomic groups, which has important implications for the generalizability of trait–environment relationships. It will also help to identify understudied traits and methodological differences such as variation in study environments (e.g., laboratory or field) or life stage definitions that require further examination (Merritt and Dixon 2011; Winkler et al. 2024). Addressing such knowledge gaps could advance our ability to design comparable and impactful studies, generalize findings, and, ultimately, apply trait‐based frameworks to seed‐based restoration effectively.

In addition to identifying knowledge gaps for taxa and traits, it is critical to understand whether and how environmental factors have been integrated into seedling trait studies. To better leverage trait data in advancing understanding of recruitment and regeneration, trait–filter frameworks have been developed to predict the long‐term success of plants in different environmental contexts (Laughlin 2014a, 2014b; Spasojevic et al. 2014; Harrison and LaForgia 2019; Funk et al., 2021; Ramachandran et al. 2023). Applied at the recruitment stage, this conceptual model emphasizes how abiotic factors, such as drought, temperature extremes, or disturbances, act as selective filters that determine seedling establishment based on their respective functional traits (Larson and Funk 2016). At the same time, the measured attributes of an individual seedling or population can vary in response to dynamic environments via phenotypic plasticity (e.g., Larson and Funk 2016b) or ontogeny (Garbowski et al. 2021; Havrilla et al. 2021), complicating trait‐based predictions of performance. A synthesis of available seedling literature can help to prioritize research efforts by identifying knowledge gaps and promising avenues for future work with respect to environmental drivers and traits at the seedling stage, whether multiple interacting drivers are considered in tandem, and where further investigation is needed to understand how trait–environment interactions shape seedling establishment and persistence. A comprehensive integration of environmental context into seedling trait studies will be essential to quantify ecological patterns in the field and improve predictive capabilities for seedling regeneration and restoration planning (Seastedt et al. 2008; Laughlin 2014a, 2014b; Schlaepfer et al. 2012; Suding et al. 2015). Unfortunately, this work is stymied not only by the lack of synthesis on the relationship between seedling traits and performance and how they may be moderated by the abiotic and biotic environment, but also on the state of the field and information available to conduct such an analysis.

To identify the knowledge gaps in seedling trait research, we created the EPiC (Early Plant information Combined) database. EPiC is a database of categorical information, including trait types, from studies conducted on seedlings of plants native to the United States with the goal of understanding how seedling traits are studied and in which contexts. We also aggregate this information due to another challenge in seedling functional ecology of a lack of consistency in how seedlings are defined by researchers across studies with regard to age or life stage, such as number of days or number of leaves (Moles and Westoby 2004a, 2004b; Leck et al. 2008). Without clear delineation of life stages, it is difficult to determine whether findings are comparable or reflect differing developmental phases, and if seedlings under‐study are adult—further propagating the research disparity. Clarifying the approaches utilized in seedling trait articles across multiple US ecosystems can inform the application of these traits in restoration strategies and ecological modeling and create a foundation to advance future research in these areas.

We conducted a systematic review of published literature to characterize the current state of seedling trait research for species native to the US. We aimed to identify methodological approaches and patterns, temporal trends, and critical knowledge gaps, guided by five central research questions: (1) *Who*: what plant species are studied at the seedling stage? (2) *What*: what traits are commonly measured? (3) *When*: at what age or developmental stages are seedlings assessed? (4) *Where*: in which US ecoregions are seedling experiments concentrated? and (5) *How*: under what biotic and abiotic conditions are seedling traits evaluated? As trait‐based ecology has increasingly emphasized predictive frameworks that link functional traits to performance outcomes (McGill et al. 2006; Funk et al. 2017), we also asked: How have temporal trends in seedling trait research shifted over time? The EPiC database, developed for this synthesis, integrates broad covariates on the abiotic and biotic drivers, seedling functional traits, and experimental contexts utilized by seedling trait researchers.

## 2 Methods

We conducted a systematic review of peer‐reviewed literature to compile a comprehensive database of seedling trait articles and qualitative covariates of these articles across diverse ecosystems in the United States (EPiC database; Figure S1). We compiled covariates from each article, and not quantitative trait data on seedlings, to describe the breadth of seedling trait research in the United States to date.

### 2.1 Literature Search

To assemble the EPiC database, we queried the ISI Web of Science (WOS) database seeking peer‐reviewed publications related to plant traits and seedling articles published between 1900 to 28 February 2024. The search terms were: (plant OR plants) AND (seedling* OR ontogen* OR “early‐life” OR “early life”) AND (function* OR trait* OR phenotyp* OR morpholog* OR physiolog* OR phenolog*) (Table S1). We excluded articles that focused on cultivated species, tree species, non‐native species relative to the US, as well as articles conducted outside of the US (Figure S1). Following this first step, we filtered the results in WOS to include the “Research Areas” of Plant sciences, Environmental sciences, Ecology, Forestry, Science technology, and relevant subtopics of Biodiversity conservation, Evolutionary biology, Developmental biology, Physiology, Anatomy and morphology, and Reproductive biology.

We removed duplicate records from the WOS search results and performed a review of the content to identify any quality control issues. We improved inter‐coder reliability by collaboratively reviewing a subset of 15 titles and abstracts, to promote consistent evaluation of the selection criteria before independent screening of the remaining articles. In addition to reviewing article alignment with the criteria mentioned above (e.g., non‐cultivated and non‐tree species), we assessed alignment with the “seedling” focus of this synthesis. We did not impose a specific age or developmental stage cutoff; instead, we relied upon authors calling the plant stage under‐study as “seedlings” (or a related term), then acquired the age or stages they deemed to be seedlings. This allowed us to capture variation in how researchers characterize seedlings ranging from age‐, stage‐, or size‐based delineations. After this alignment, eight of the authors independently screened approximately 20% of the titles and abstracts to calibrate criteria and train an AI‐assisted screening tool. We utilized the AI‐powered systematic review tool Rayyan (Rayyan Systems Inc., Qatar; Ouzzani et al. 2023) to assist with screening and classification of relevant literature following standard protocols detailed in Valizadeh et al. (2022). Rayyan provided recommendations for inclusion or exclusion of the remaining 80% of articles by assessing titles and abstracts. To verify the accuracy and reliability of these recommendations, we manually screened an additional 20% of the records labeled by Rayyan, confirming agreement and consistency with our selection criteria (*sensu* Foo et al. 2021). Following this AI‐assisted title and abstract screening, eight of the authors further excluded unsuitable articles via full‐text screening, resulting in a refined dataset suitable for detailed review.

The final dataset consisted of 181 articles (i.e., articles; Figure S1). We extracted qualitative data on the following variables from each article: study species, intraspecific variation (whether the experiment included variation among populations, cultivars, varieties), research setting (field, greenhouse, or growth chamber), manipulation of abiotic and biotic factors, growth media, nutrient applications, seedling age and age units, measured traits and their units, and GPS coordinates for field experiments (see Johnson 2025). Study species were cross‐referenced with the United States Department of Agriculture (USDA) Plants Database to assign functional groups and life‐history strategies. Trait names were extracted and manually categorized into trait types (morphological, physiological, phenological, performance/growth), as well as by plant anatomical focus (aboveground, belowground, or whole plant). We categorized abiotic treatments into eight classes based on the type of environmental factor altered in each experiment: water (e.g., drought, irrigation), disturbance (e.g., fire, grazing), temperature (e.g., warming, cold exposure), salinity (e.g., salt concentration in soil or water), pollutants (e.g., chemical contamination, herbicide application), nutrients (e.g., nitrogen, phosphorus availability), substrate (e.g., soil texture, composition, or structure), and light (e.g., shading, altered photoperiods).

Using these data, we summarized patterns across articles based on five key methodological aspects aligned with our research questions: (1) study species, including their functional groups and life‐history strategies; (2) traits, classified into multiple categories; (3) seedling age at measurement; (4) geographic location and ecoregion; and (5) experimental manipulations of abiotic and biotic conditions. Spatial data were processed and mapped using the *sf* (Pebesma 2018), *terra* (Hijmans 2024), and *raster* (Hijmans 2023) packages in R, with climate data obtained via geodata (Hijmans 2025). We included scale bars and north arrows on the maps using *ggspatial* (Dunnington 2025). Experiment locations were also classified within Whittaker biome space using the *plotbiomes* package (Figure S2; Ștefan and Levin 2018) to contextualize the climatic conditions under which seedling traits were measured.

### 2.2 Statistical Analysis of Temporal Trends

We conducted all statistical analyses in R (R Core Team 2023 v4.2). We used generalized linear models (GLMs) with a Poisson error distribution to test whether the number of articles measuring different trait attributes changed across years. These models analyze counts of studies/articles per year—reflecting trends in research focus and publication frequency—rather than quantitative seedling trait values themselves, consistent with the covariate‐level synthesis approach described above. These models assess trends in the number of unique articles per year associated with each trait category (article ~ year * trait category; with trait category as performance/growth, morphological, physiology, and phenological). This model aims to assess if the frequency of published articles reporting on seedlings with trait evaluations has changed with time, to assess shifts in focus from research of performance and morphological traits (i.e., outcomes) to those looking at physiological or phenological traits (predictors or mechanisms driving outcomes). We also tested if trait type tested differed with time (article ~ year * trait type; with trait type categorized as aboveground, belowground, or whole plant), as well as functional group (article ~ year * functional group; with functional group as graminoids, forbs/herbs, shrubs). To achieve this, we created summary tables that counted the number of unique articles per year as an ordered factor for each category level. In addition, to more directly capture shifts in which ecological and functional trait covariates were recorded over time, we conducted a complementary analysis quantifying the number of studies/articles reporting each covariate category per year, allowing us to distinguish changes in overall publication volume from changes in the relative frequency with which specific covariates were measured. Lastly, we assessed model fit by checking for overdispersion using residual diagnostic plots and dispersion statistics and examined the significance of interaction terms using the *anova* function to determine whether temporal changes in article frequency varied across factor levels.

### 2.3 Phylogenetic Tree

We constructed a phylogenetic tree of plant species identified in this systematic seedling trait synthesis using the *V.PhyloMaker2* R package (Jin and Qian 2022). Species names were cleaned and standardized to resolve taxonomic synonyms in accordance with the USDA Plants database (version 18.2, December 2023). For each unique species, we obtained genus and family‐level classifications using the *taxize* package (Chamberlain and Szöcs 2013), with manual corrections applied where necessary due to incomplete matches or hybrid taxa. We used the *phylo.maker* function in *V.PhyloMaker2* (Jin and Qian 2022) to generate a phylogeny based on mega‐trees developed by Zanne et al. (2014) and Smith and Brown (2018), with the “S3” argument specified to ensure maximal species inclusion by binding unmatched taxa at the genus or family level. We visualized the resulting tree using *ggtree* (Yu et al. 2017). Additional covariates, including plant family (for common families with more than six species) and the number of articles in which each species appeared, were mapped onto the tree to assess representation patterns across lineages. We also estimated Faith's phylogenetic diversity to quantify the total evolutionary lineage represented by identified species (*picante* package in R, Kembel et al. 2010). We observed heavy skew in the degree of representation (i.e., number of studies) among identified species, and consequently, focus on descriptive assessments of relative representation rather than quantitative tests (e.g., phylogenetic signal).

## 3 Results

### 3.1 Search Results

We found 4607 articles during our WOS search, which was filtered to 181 suitable articles published between 1974 and February 2024.

### 3.2 Who: Species Classification, Trait Types, and Trait Categories

**FIGURE 1:**
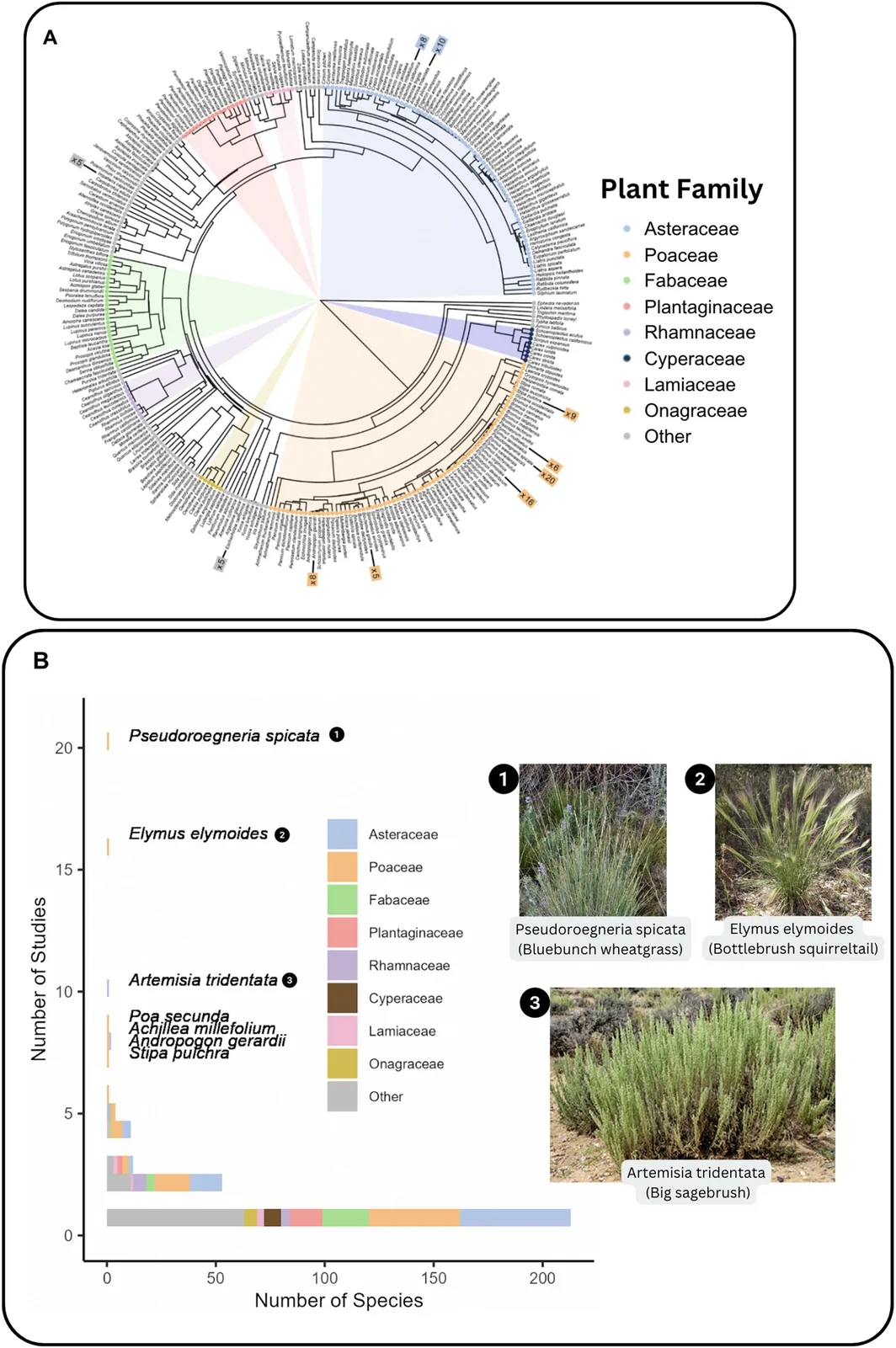
(A) Phylogenetic tree of native herbaceous and shrub species studied as seedlings within the United States. Colored circles and shaded portions correspond with plant families. Labels on the outside of the tree indicate species represented in five or more articles. (B) Histogram of seedling species included in this review and photographs of the three species ( *Pseudoroegneria spicata* , *Elymus elymoides* , *Artemisia tridentata* ) most frequently studied at the seedling stage. 26% of the families make up the other category ranging from 1 to 5 species per family. Photos (left to right): Plant Diversity, USFWS Mountain‐Prairie, USFWS Mountain‐Prairie.

A total of 304,302 species belonging to 48 unique families were identified in our dataset (Figure 1A), representing 8939 Myr of cumulative evolutionary history (Faith's PD = 8939.1 Myr). Graminoids and forbs dominated the functional group composition of species within the dataset (38% and 44% of species cases tallied by unique combination of species × article, respectively; Figure 4D). Similarly, two plant families—Asteraceae and *Poaceae—accounted* for 24% and 36% of species cases, respectively, while dozens of families were represented by just a few species cases (Figure 1B). Even within commonly represented families, however, a small subset of species tended to be over‐represented, including *Pseudoroegneria spicata* (Poaceae), *Elymus elymoides* (Poaceae), and *Artemisia tridentata* (*Asteraceae*), which accounted for 9% of species cases (Figure 1B). Lastly, intraspecific variability was explicitly measured or considered in 63% of articles (e.g., via the inclusion of multiple populations or varieties per species).

### 3.3 What: Describing Evaluated Seedling Traits

**FIGURE 2:**
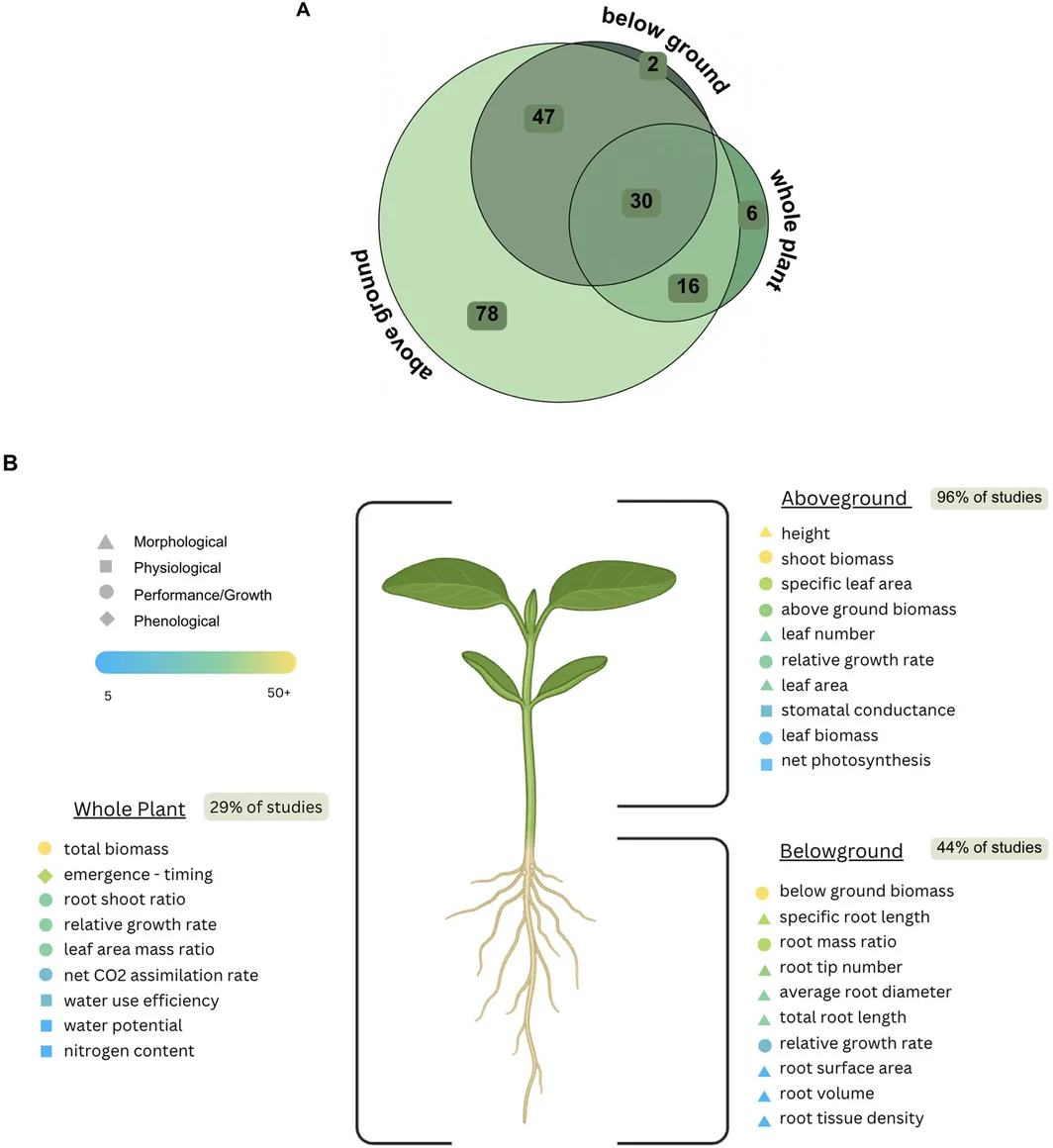
(A) Euler diagram depicting the overlap of articles measuring trait types (e.g., whole plant, above ground, and below ground). (B) Illustrative summary of the ten most frequently measured seedling traits in articles included in the systematic review by trait type. The shape next to each trait name indicates the classification of trait category (e.g., morphological, physiological, performance/growth, or phenological) and the shape color gradient ranging from blue to yellow corresponds to the number of articles that measured each trait ranging from low to high respectively.

Across all 181 articles, we identified 298 seedling traits after consolidating traits with name variations (Johnson 2025; a list of data sources used in this systematic review are provided in the Data sources section). The ten most commonly reported traits were total biomass (49 articles), height (48 articles), belowground biomass (46 articles), emergence date (36 articles), mean shoot biomass (35 articles), specific leaf area (27 articles), aboveground biomass (23 articles), root‐to‐shoot ratio (22 articles), leaf number (21 articles), and specific root length (21 articles). Most articles (80%) measured multiple trait types. Aboveground traits were assessed most frequently (96% of articles), followed by belowground traits (44% of articles) and whole‐plant traits (29% of articles) (Figure 2). Across functional trait categories, morphological traits (36%), and performance or growth traits (34%) were most studied, whereas physiological traits (15%) and phenological traits (13%) remained underrepresented (Figure 2). Relationships between trait types were visualized using circular plots created with the *circlize* package (Gu et al. 2014).

### 3.4 When: Evaluating Seedling Age and Stage of Trait Measurements

**FIGURE 3:**
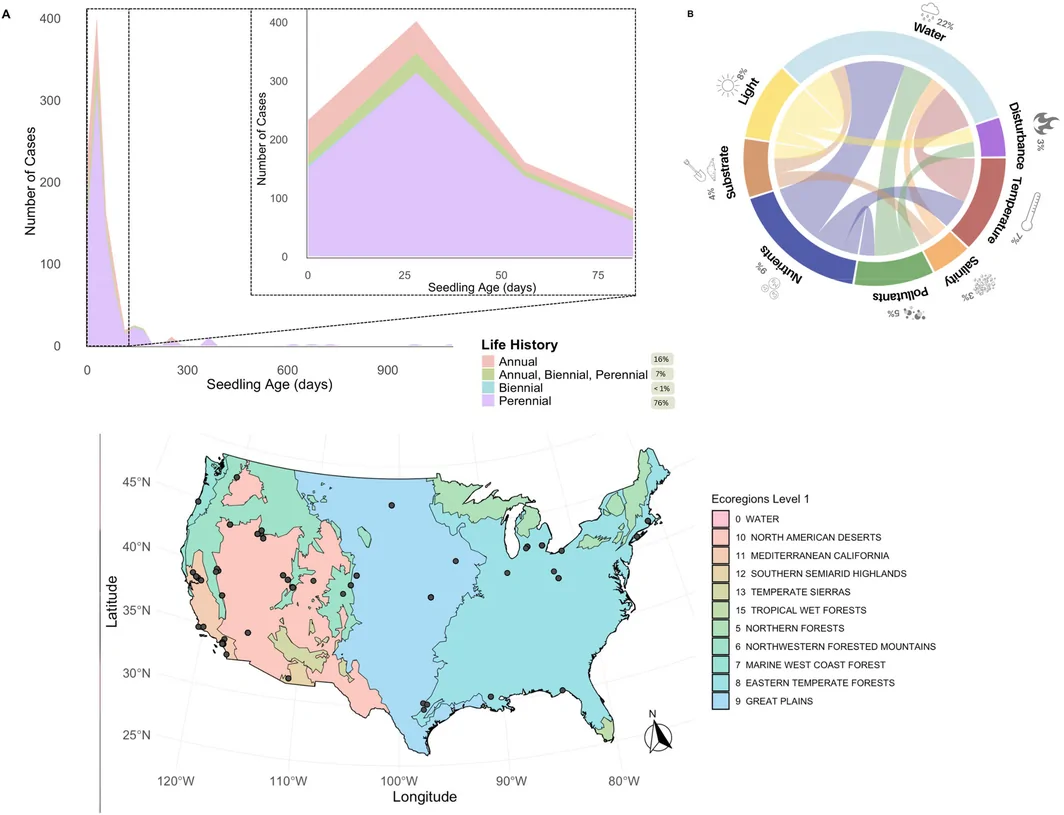
(A) Area plots summarizing raw data trends of a stacked bar chart for the number of cases of unique articles, species, and age combinations (*n* = 983). The full range of seedling ages recorded numerically is converted to days and categorized by life history. The inset consists of seedling ages for the first 90 days. (B) Chord diagram displaying the inter‐relationships between abiotic manipulations within experimental articles. This diagram includes only articles that manipulated more than one abiotic factor; articles manipulating a single abiotic factor are not represented here. The length of each arc corresponds with the total number of articles investigating that abiotic factor and the thickness of the chord corresponds with the number of articles for which that abiotic relationship was investigated. The percentages reflect unique articles by abiotic factor (C) Distribution of field experiment sites across level 1 Ecoregions within the contiguous United States. This excludes articles conducted in Hawaii.

Researchers largely assessed seedlings by numeric age (87% of articles) as opposed to life stage or size metrics, such as height class or first true leaf (13% of articles). Of the articles that reported seedling age numerically, most measurements were collected at relatively young ages: seedlings younger than 90 days represented approximately 76% of articles, while fewer articles (24%) took measurements beyond this point at up to 3 years of age (Figure 3A). Only 2% of articles measured seedling traits over 1 year old, and of those articles, 66% were shrub species.

### 3.5 Where: Geographic Distributions of Seedling Trait Field Experiments

Seedling studies exhibited an uneven geographic distribution, with substantial concentration in arid and semi‐arid regions (57% of articles) such as the North American deserts and Mediterranean California Ecoregions (Figure 3; Omernik and Griffith 2014).

### 3.6 How—Experimental Manipulations and Conditions

Most studies were experimental manipulations to track age and stage, and across the dataset, both abiotic and biotic manipulations were unevenly represented. Abiotic factors were most commonly manipulated (66% of articles). Within this subset, articles focused on water stress (39%) and nutrient stress (18%) were most common, with other factors such as substrate type (7%) and disturbance regimes (5%) tested far less frequently. Additionally, 8% of articles included multiple abiotic treatments (Figure 3B). In contrast, biotic factors were manipulated in only 22% of articles. Of these, soil feedback was the most common (15% of all articles), while herbivory (3%) and pollination (< 1%) were much less frequently studied. For experiment environments, 56% of experiments were conducted in greenhouses, 28% in the field, 14% in growth chambers, and fewer than 1% across multiple environments. Regarding growth media, 66% of experiments used loam soil, 9% used sand, and 23% used a mix which included sand, soil, and/or perlite.

### 3.7 Temporal Patterns of Seedling Studies

**FIGURE 4:**
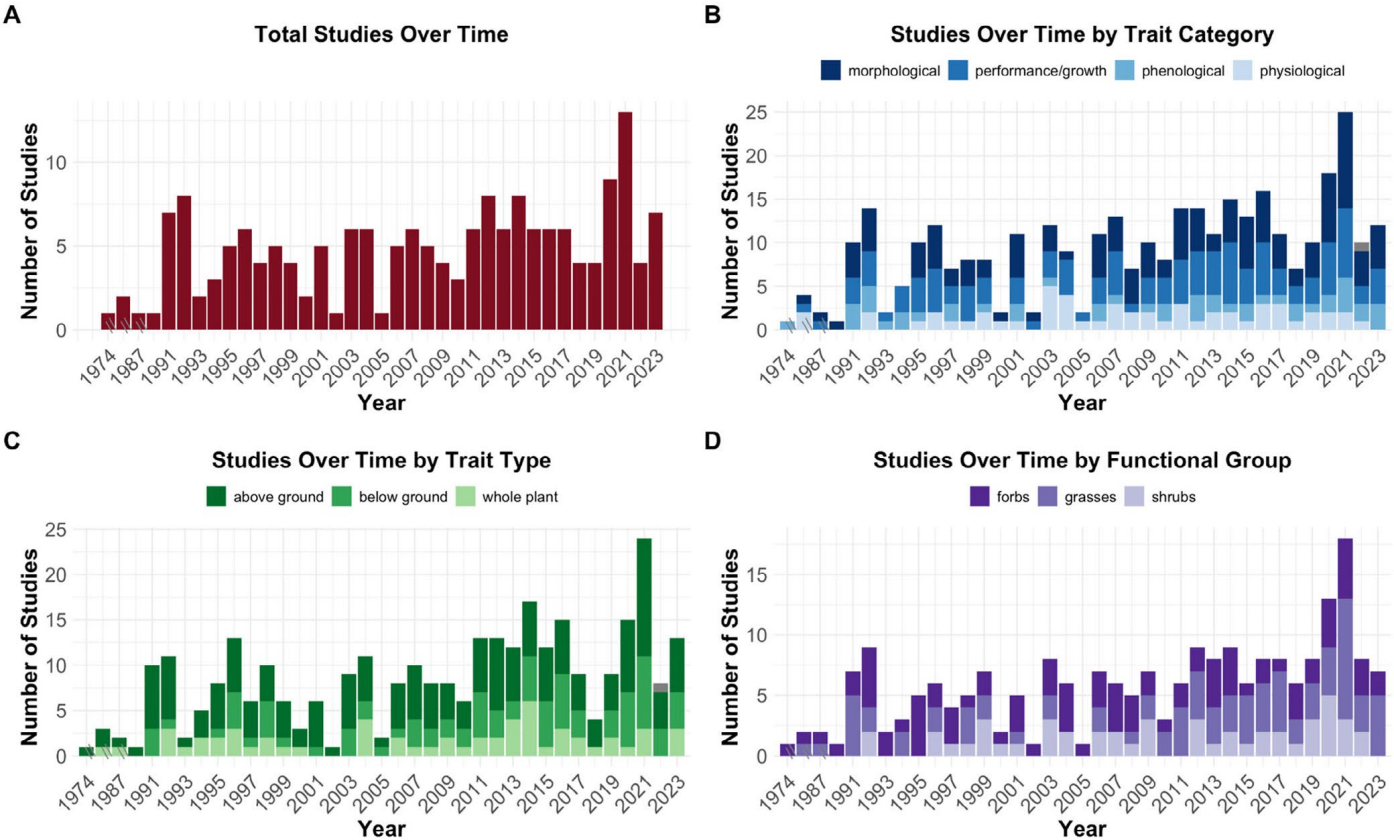
Seedling articles included in the EPiC database, published over time as it relates to plant traits and functional groups. (A) Total number of seedling articles in the US through 2023. (B) Trait category occurrences (performance/growth, morphological, physiology, and phenological) measured in articles (articles × trait category) over time. (C) Trait type occurrences (above ground, below ground, and whole plant) measured in articles (articles x trait type) over time. (D) Seedling functional group occurrences (forbs/herbs, graminoids, and shrubs) measured in articles (articles × functional group) over time.

The number of seedling articles in the United States significantly increased over time (*z* = 3.13, *p* < 0.001), with a rise in publications beginning in 2012 and the most seedling publications to‐date occurring in 2021 (Figure 4). The frequency with which different trait categories, trait types, and functional groups changed through time was not statistically significant (Table S2). Similarly, temporal trends in covariate reporting frequency were not statistically significant for any abiotic factor; however, studies reporting water manipulation showed an increasing, though non‐significant, trend over time.

## 4 Discussion

This study provides a critical synthesis of how seedling traits have been studied across the United States over the past 5 decades. By systematically reviewing this body of work, we reveal key patterns in researcher decisions related to trait choice, species focus, and geographic coverage—highlighting where researchers have concentrated efforts within seedling ecology and where major gaps remain. These findings expose a disconnect between the established ecological importance of the seedling stage (Moles and Westoby 2004a, 2004b) and what and how often seedling traits are studied. An increasing number of articles highlight the growing relevance of trait‐based seedling ecology, and here we reveal opportunities to expand research on traits related to belowground functioning and phenology, better understand the influences of seedling age and biotic interactions, and expand seedling ecology studies beyond arid ecosystems.

### 4.1 Who—Species Studied at the Seedling Stage

Articles included a diverse range of taxa from nearly 50 plant families, although nearly half of study species belonged to Asteraceae and Poaceae families. These are among the most species‐rich plant families worldwide (Christenhusz and Byng 2016) and comprise a meaningful portion of many North American plant communities. Our synthesis also revealed pronounced taxonomic biases within the represented families, in which species such as *Pseudoroegneria spicata* (Poaceae), *Elymus elymoides* (Poaceae), and *Artemisia tridentata* (Asteraceae) were disproportionately represented in seedling trait articles. These are priority restoration species for habitat creation in North American drylands (Doherty et al. 2024) for post fire re‐seeding of millions of hectares of land often with grasses (Wood et al. 2015) and widespread occurrence across these ecosystems and inclusion within seed mixes for large‐scale restoration (Ladouceur et al. 2018; Kilkenny et al. 2023; Davies et al. 2025). Furthermore, the prevalence of Asteraceae, specifically *Artemisia tridentata* , is likely driven by being a major target for restoration efforts in the western US to conserve Greater Sage‐Grouse habitat (Pyke 2011) and faces seedling establishment issues (Landeen et al. 2026). We encourage future work to move beyond commonly used restoration species, although the focus on priority species may reflect funding or applied needs (e.g., the National Native Seed Needs Assessment). Broadening the taxonomic scope of seedling articles to include diverse functional groups adapted to different climates can strengthen the restoration toolkit and improve the adaptive capacity of restored systems (Funk et al. 2024).

### 4.2 What: Work Has Focused on Easily Measured Aboveground Traits

Articles mainly focused on morphological traits, particularly aboveground traits including plant height, leaf area, and aboveground biomass. This pattern likely reflects logistical constraints in which aboveground traits are simpler and faster to measure than root or physiological traits, which often require destructive sampling and specialized equipment (Pérez‐Harguindeguy et al. 2013; Freschet, Pagès, et al. 2021; Freschet, Roumet, et al. 2021). Furthermore, performance traits (e.g., biomass, height) are direct measures of performance and are also easy to understand and are management‐relevant (Gornish et al. 2023). In contrast, belowground traits were among the least studied, despite their fundamental role in determining seedling success under environmental stress and interactions (Foxx and Kramer 2020). Root architecture and interactions with soil microbiota are vital for water and nutrient acquisition, interactions, stress tolerance, and overall establishment (Bardgett et al. 2014; Foxx and Kramer 2020; Freschet, Pagès, et al. 2021; Freschet, Roumet, et al. 2021; Larson, Anacker, et al. 2020; Larson et al. 2020). Therefore, the omission of these traits limits our ability to account for whole‐plant responses to abiotic and biotic conditions during seedling establishment, though new resources and technology have made it easier to assess root traits (Ahler et al. 2025). While seedling roots are easier and faster to analyze than adult plants, when extracting from soil and cleaning, their small biomass can present a challenge without the right equipment (i.e., a microbalance)—particularly if researches target the youngest seedling stages of less than a few weeks old as was prevalent in our dataset (Figure 4a).

Researchers examined phenological and physiological traits such as seedling emergence timing or photosynthetic ability less frequently than morphological traits. Phenological traits hold considerable promise for guiding restoration timing and design and understanding community assembly—particularly through mechanisms like priority effects, germination cues, and interspecific competition (Davies et al. 2013; Larson et al. 2021). Complicating trait assessments, seedling trait values can change in response to competition and other factors, with downstream consequences for later growth and species interactions (Schwinning et al. 2017; Foxx 2021). Physiological traits also may face challenges moving forward; similar to root traits, small size represents a logistical constraint, though emerging technologies such as the LI‐COR (LI‐COR, USA) small plant chamber and Walz Mini‐PAM fluorometers (WALZ, Germany) offer solutions that have yet to be widely incorporated into seedling trait research. We also observed a wide range of physiological trait metrics reported in the literature, with limited replication across articles, hindering cross‐article comparisons and synthesis. For example, 102 unique physiological traits were captured in our synthesis but only 20 of those were studied in two or more articles. Given the wide breadth of unique traits reported in all trait categories, there is still a critical need to develop consensus on a standardized set of high‐priority, functionally relevant traits for application in basic and applied seedling research (Winkler et al. 2024).

### 4.3 When—A Clearer Definition of “Seedling” Is Needed to Unify Seedling Trait Research

Our synthesis also revealed substantial variation in how researchers defined and measured the seedling stage, which complicates cross‐study quantitative analyses of trait data. While most articles (76%) focused on individuals younger than 3 months, some included plants aged well beyond this age window, up to 3 years, while still referring to them as seedlings. Because the seedling phase is characterized by rapid morphological and physiological changes (Poorter et al. 2012), inconsistent use of the term “seedling” across articles may obscure important trait–function relationships. This inconsistency highlights a key conceptual gap: the lack of a clear and consistent definition of the seedling stage in trait‐based research. Winkler et al. (2024) noted that seedlings are typically defined as life stages in which the developing plant still relies on seed‐derived resources (e.g., endosperm or cotyledons). From this perspective, researchers measuring traits in older plants may inadvertently conflate traits of plants at seedling and juvenile life stages. Moreover, stage‐based categorizations (e.g., cotyledon stage, first true leaves, multiple true leaves) provide more biologically meaningful distinctions than age alone, particularly given that some fast‐developing species can reach reproductive maturity while still morphologically resembling seedlings. Given that plant traits can shift rapidly through ontogeny (Garbowski et al. 2021; Havrilla et al. 2021), such inconsistencies risk introducing noise into syntheses and comparisons, as traits expressed in true seedlings versus juveniles are likely governed by different resource limitations and environmental interactions (Lawrence‐Paul et al. 2023). We recommend adopting ontogenetically grounded criteria to define seedling stages more precisely (Baskin and Baskin 2014), which would enhance the consistency and ecological relevance of future seedling trait‐based work. During our synthesis, we encountered several studies with insufficient methodological detail for us to determine seedling age, which should be included. Ultimately, clarifying definitions and promoting greater consistency in how the seedling stage is defined and measured would enhance the interpretability of trait‐based research and support the development of more robust predictive models for seedling performance across environments (Kitajima and Fenner 2000; Mason et al. 2013).

### 4.4 Where: Seedling Studies Are Largely Focused on US Dryland Regions

Most studies were conducted in arid and semi‐arid regions of the western United States, likely due to water stress being a major bottleneck for seedling survival. Reports in dry ecosystems find that of 2% (243 of 12,150) of shrub seedlings surviving summer drought (León et al. 2011) and 12% probability of cool‐season grasses transitioning from germinated seeds to emerged seedlings in the arid Western US (James et al. 2011). Because dry ecosystems are often prioritized for seed‐based restoration in the United States due to large‐scale degradation (Oldfield and Olwell 2015), seedling trait research is concentrated in these ecosystems and is especially valuable to help identify trait values that may promote seedling survival. For example, research to date has shown the importance of root traits, including root depth and root fibrosity, for resource acquisition, competition, and survival (León et al. 2011; Foxx and Kramer 2020; Garbowski et al. 2020). These patterns may arise in‐part due to the spatial filter we imposed, and the context‐dependent and US‐centric focus bounds our interpretation to this region, and expanding regions will help identify broader patterns in seedling strategies that encompass results from other ecologically important regions.

Notably underrepresented are mesic and temperate ecosystems—such as eastern deciduous forests, wetlands, and montane habitats—where seedling dynamics may differ substantially due to variation in climate, soil conditions, and species composition. Research in these ecosystems could reveal different seedling strategies than those documented in water‐limited environments, particularly regarding traits related to light competition, which can be a major limiting factor for seedling establishment and survival in forest understories (Kunstler et al. 2016; Howard and Goldberg 2001). Additionally, pathogen pressure is predicted to be higher in warm and moist environments compared to dry or well‐drained environments (Dalling et al. 2011), suggesting that seedling traits related to pathogen resistance may be more critical in mesic systems. Moreover, as restoration efforts increasingly expand to degraded eastern temperate forests (Stanturf et al. 2014; Vitt et al. 2010), understanding seedling functional traits of herbaceous understory plants in these ecosystems becomes critical for improving restoration outcomes.

### 4.5 How: Past Work Focused on Trait Responses to Water and Nutrient Availability While Other Environmental Drivers Remain Understudied

Studies of seedling trait variation have mainly focused on responses to drought or variable nutrient availability. In contrast, trait variation and response to additional ecologically relevant factors remain relatively underexplored. Among disturbance regimes, fire and grazing have been studied to some extent, but trampling, flooding, burial, frost heaving, and mechanical damage from wind or animals are rarely investigated despite their importance in many ecosystems. Temperature extremes, pollutants (e.g., heavy metals, nitrogen deposition), and biotic interactions beyond pairwise competition (e.g., facilitation, allelopathy, plant–soil feedbacks) similarly warrant greater attention in seedling trait research. This pattern likely reflects both the comparative ease of manipulating water and nutrient levels in controlled environments (Moles et al. 2011; Sack and Buckley 2016) and the emphasis of many studies on restoration in arid and semi‐arid regions, where drought stress is a primary concern (Oldfield and Olwell 2015; Leger and Baughman 2015). However, trampling from grazing livestock or recreational use can cause substantial seedling mortality and warrant further study as morphological traits such as prostrate growth form, shorter stature, and increased stem flexibility may confer trampling resistance (Cole 1995; Pescott and Stewart 2014). Similarly, seedling responses to flooding are increasingly important as climate change intensifies precipitation extremes; traits such as aerenchyma formation and adventitious root development determine flooding tolerance and may predict species' persistence in wetlands and riparian zones (Colmer and Voesenek 2009; Yamauchi et al. 2018).

Biotic interactions, such as plant–soil feedbacks and herbivory, have also received comparatively less attention in studies of seedling trait variation. Herbivory is important because it can cause differential seedling mortality across species and alter competitive outcomes, with seedling herbivory representing the primary source of mortality for most seedlings (Hanley and Sykes 2009; Moles and Westoby 2004a, 2004b). Similarly, plant–soil feedbacks, including those altered by invasive species, can create recruitment barriers for native seedlings, with invasive species often modifying soil microbial communities in ways that favor their own establishment while inhibiting native seedling performance (Al‐Khayri et al. 2024; Verbeek and Kotanen 2019). Traits mediating resistance to herbivores, tolerance to pathogens, and response to soil biota may be as important as abiotic stress tolerance for predicting establishment success in many ecosystems and require greater study in the US.

Beyond targeted abiotic and biotic treatments, experimental methods and settings can further constrain comparability across varied experiment settings (e.g., from laboratory to field patterns). Most studies were conducted in controlled environments allowing for higher throughput of trait measurements with more repeatability and comparability across studies. Field‐based studies remain comparatively limited, even though they are essential for capturing the ecological realism of trait–environment relationships (Chambers et al. 2017; Johnson et al. 2022). Furthermore, among studies that examined multiple factors simultaneously, water and nutrient manipulation were most common, followed by water and temperature combinations. These multi‐factor experiments can reveal important interactive effects that would be missed when studying factors in isolation. For example, Valliere (2019) demonstrated that native California coastal sage scrub seedlings responded positively to nitrogen and water additions when grown alone but performed best under low‐resource conditions when competing with invasive species—a finding that shifts our understanding of how resource availability affects restoration outcomes in invaded systems.

### 4.6 Limitations and Future Research Directions

This synthesis underscores progress in seedling trait research and highlights several gaps in the current literature, though there are several important limits of our systematic review. Notably, the EPiC database focuses on US ecoregions without an ability to extrapolate globally or the ability to incorporate the robust global seedling research. Additionally, our inclusion criteria, which required published trait data, may have biased the dataset toward formal experiments, potentially excluding valuable insight from field observations, management reports, practitioner‐generated data, and other gray literature. Relevant data deposited in unpublished studies or data repositories without a peer‐reviewed journal article were not incorporated into this synthesis (see Culina et al. 2018). At the same time, our review provides important insight into the traits, taxa, geographic regions and methods that have been focal points for seedling trait researchers across the biodiverse US.

Looking forward, advancements are needed to address several fundamental and integrative questions that emerged from this synthesis. A critical gap highlighted by this synthesis is our limited understanding of what differentiates the seedling stage from later stages in terms of trait expression, functional performance, and environmental vulnerability. As plants mature, shifts in resource allocation and functional strategies reduce vulnerability and alter how traits contribute to overall performance and survival (Barton 2024). Cross‐stage studies are needed to understand how seedlings respond to stressors—and whether specific traits or abiotic factors are disproportionately important during this early life stage—in order to refine our understanding of regeneration dynamics and possible restoration strategies. For example, traits related to emergence timing or stress tolerance may play a critical role in early establishment from seed but may become less influential at later stages (Kempel et al. 2023).

Another priority is the identification of traits that are most ecologically and practically informative for predicting seedling performance under varied or complex environmental contexts (Funk et al. 2024). Restoration practitioners face practical constraints, and clearer guidance on trait selection—grounded in both ecological understanding and applied outcomes—would improve the relevance and utility of trait‐based approaches (Merchant et al. 2023, *but see* Gornish et al. 2023). Given the wide breadth of unique traits measured by seedling trait researchers to date, coordinated efforts to prioritize core trait sets, and to understand how these traits perform under different stressors and across life stages, will be critical next steps.

## 5 Conclusion

Our review synthesized the breadth of seedling trait studies in the US and highlights well‐studied areas and gaps in methodology, taxonomy, and geography that shape the field. It also highlights the untapped potential of seedling trait data to inform more effective, ecologically grounded research and restoration strategies, by pinpointing key research gaps—particularly in underrepresented trait categories (e.g., belowground and phenological traits), functional groups, ecoregions, and experimental designs—this synthesis outlines clear directions for advancing seedling ecology in a rapidly changing world. Efforts to improve open access to seedling trait data, such as through the EPiC database, will further catalyze progress by enabling cross‐article comparisons and meta‐analyses that improve standardizing data sources and assessing traits that greatly affect seedling functioning and ecology. Ultimately, building a more coordinated seedling trait field will strengthen our capacity to support biodiversity, enhance ecosystem services, and build resilience in the face of accelerating environmental change.

## Author Contributions

**Nia M. Johnson:** conceptualization (equal), data curation (lead), formal analysis (lead), investigation (equal), methodology (equal), project administration (equal), supervision (lead), visualization (lead), writing – original draft (lead), writing – review and editing (equal). **Leah J. Lenzo:** conceptualization (equal), data curation (equal), funding acquisition (equal), investigation (equal), methodology (equal). **Amelia G. Renner:** data curation (equal), investigation (equal), writing – original draft (equal). **Sofia M. A. Al‐Shayeb:** data curation (equal), investigation (equal), writing – original draft (equal). **Julie E. Larson:** conceptualization (equal), data curation (equal), funding acquisition (equal), investigation (equal), methodology (equal), writing – review and editing (equal). **Magda Garbowski:** conceptualization (equal), data curation (equal), funding acquisition (equal), investigation (equal), methodology (equal), writing – review and editing (equal). **Mandy L. Slate:** conceptualization (equal), data curation (equal), funding acquisition (equal), investigation (equal), methodology (equal), writing – review and editing (equal). **Agneray C. Alison:** conceptualization (equal), funding acquisition (equal), investigation (equal), methodology (equal). **Sarah C. Barga:** conceptualization (equal), funding acquisition (equal), investigation (equal), methodology (equal), visualization (supporting). **Tayah C. Carlisle:** data curation (supporting), investigation (equal). **Andrew Currens:** data curation (supporting). **McKenzie Dorrell:** data curation (supporting). **Caroline A. Havrilla:** conceptualization (equal), funding acquisition (equal), investigation (equal), methodology (equal). **Marina L. LaForgia:** data curation (supporting), funding acquisition (supporting), investigation (supporting), methodology (supporting). **Amanda Paradis:** data curation (supporting). **Moira M. Newman:** data curation (supporting). **Samantha Rosa:** investigation (supporting), writing – original draft (supporting). **Michaela Sershen:** data curation (supporting). **Daniel E. Winkler:** conceptualization (equal), data curation (equal), funding acquisition (equal), investigation (equal), methodology (equal), writing – review and editing (supporting). **Halo Wood:** data curation (supporting). **Alicia J. Foxx:** data curation (equal), funding acquisition (lead), investigation (equal), methodology (equal), project administration (equal), supervision (supporting), writing – review and editing (equal).

## Funding

This research was supported by The Chicago Botanic Garden's Synthesis Center for Conservation and Restoration (sCORE) and The United States’ Bureau of Land Management Plant Conservation and Restoration Program.

## Conflicts of Interest

The authors declare no conflicts of interest.

## Supporting information


**Figure S1:** PRISMA 2020 flow diagram for EPiC systematic reviews adapted from Page MJ, et al. 2021.
**Figure S2:** Distribution of seedling field study sites according to temperature and precipitation. Point shape indicates the classification of trait categories measured (e.g., morphological, physiological, performance/growth, or phenological) and size indicates the number of times that trait category was measured. The ellipsis highlights where the points are congregated and do not represent an analytical test.
**Table S1:** Search terms used in the scholarly literature search for publications in EPIC.
**Table S2:** Results from generalized linear models (GLMs) with a Poisson error distribution testing the number of studies measuring across years. Z and *p*‐values are presented for each trait category (performance/growth, morphological, physiology, and phenological), trait type (above ground, below ground, and whole plant), and functional group (graminoids, forbs/herbs, shrubs).

## Data Availability

The dataset underlying this article are available in Mendeley Data at https://doi.org/10.17632/7tj74kxyr8.1.
