# Correction to “From Data to Discovery: Characterizing the Scope and Limitations of Seedling Trait Studies in the U.S. With the EPiC Database”

**DOI:** 10.1002/ece3.74589

**Published:** 2026-10-22

Johnson, N. M. , L. J. Lenzo , A. G. Renner , et al. 2026. “From Data to Discovery: Characterizing the Scope and Limitations of Seedling Trait Studies in the U.S. With the EPiC Database.” Ecology and Evolution 16, no. 9: e74132. 10.1002/ece3.74132.PMC1357550742746162

The Bibliographic information of reference Al‐Khayri et al. (2024) was incorrect and should be updated with the following reference:

Ali, H. E. , A. M. Al‐Wahaibi , and M. S. Shahid 2024. “Plant–Soil Feedback and Plant Invasion: Effect of Soil Conditioning on Native and Invasive *Prosopis* Species Using the Plant Functional Trait Approach.” Frontiers in Plant Science 15: 1321950. 10.3389/fpls.2024.1321950.PMC1082483238292912

The corresponding in‐text citation in section 4.5 should be updated as follows:

Similarly, plant–soil feedbacks, including those altered by invasive species, can create recruitment barriers for native seedlings, with invasive species often modifying soil microbial communities in ways that favor their own establishment while inhibiting native seedling performance (Ali et al. 2024; Verbeek and Kotanen 2019).

We apologize for this error.

