# Supplementary material for: From Data to Discovery: Characterizing the Scope and Limitations of Seedling Trait Studies in the U.S. With the EPiC Database
Source: Ecol Evol. 2026 Sep 14;16(9):e74132. doi: 10.1002/ece3.74132 (PMC13575507; doi:10.1002/ece3.74132)
Supplement: Supplementary file 1 [file ECE3-16-e74132-s001.docx]

**Title*:*** From data to discovery: characterizing the scope and limitations of seedling trait studies in the U.S. with the EPiC Database

Nia M Johnson^1,2^, Leah J Lenzo^3^, Amelia G Renner^2^, Sofia M. A. Al-Shayeb^2,4^, Julie E Larson^5^, Magda Garbowski^6^, Mandy L Slate^7^, Agneray C Alison^8^, Sarah C Barga^9^, Tayah C Carlisle^2,10^, Andrew Currens^7^, McKenzie Dorrell^7^, Caroline A Havrilla^11^, Marina L LaForgia^12^, Amanda Paradis^7^, Moira M Newman^13^, Samantha Rosa^14^, Michaela Sershen^7^, Daniel E Winkler^15^, Halo Wood^7^, Alicia J Foxx^2,10^

^1^The Nature Conservancy, 4245 North Fairfax Drive, Suite 100, Arlington, VA, 22203, USA, ^2^Chicago Botanic Garden, Negaunee Institute for Plant Conservation Science and Action, 1000 Lake Cook Rd., Glencoe, IL, 60022, USA, ^3^University of Nevada, Reno, 1664 N. Virginia Street, Reno, NV, 89557, USA, ^4^University of Illinois at Chicago, Department of Biological Sciences, 845 W Taylor St, Chicago, IL, 60022, USA, ^5^University of Washington, School of Environmental and Forest Sciences, 3501 NE 41st St, Seattle, WA, 98195, USA, ^6^New Mexico State University, Department of Animal and Range Sciences, 2980 S. Espina Sr, Las Cruces, NM, 88003, USA, ^7^Ohio State University, Department of Evolution, Ecology, and Organismal Biology, 318 W. 12th St, Columbus, OH, 43210, USA, ^8^University of Nevada, Reno, Biology, 1664 N. Virginia Street, Reno, NV, 89557, USA, ^9^USDA Forest Service, Rocky Mountain Research Station, 1770 Kitty Hawk Drive, Cedar City, UT, 84721, USA, ^10^Northwestern University, Plant Biology and Conservation, 2145 Sheridan Rd., Evanston, IL, 60208, USA, ^11^Colorado State University, Department of Forest and Rangeland Stewardship, 1472 Campus Delivery, Fort Collins, CO, 80521, USA, ^12^Sacramento State University, Biological Sciences, 6000 J Street, Sacramento, CA, 95819, USA, ^13^Amherst College, Biology, 220 S Pleasant St, Amherst, MA, 1002, USA, ^14^University of Maryland, Department of Biological Sciences, 1204 Biology-Psychology Building, College Park, MA, 20742, USA, ^15^U.S. Geological Survey, Southwest Biological Science Center, 520 N. Park Ave., Tucson, AZ, 85719, USA

Supplemental Figures

**SI Figure 1.** PRISMA 2020 flow diagram for EPiCsystematic reviews adapted from Page MJ, et al. 2021.

**SI Figure 2.** Distribution of seedling field study sites according to temperature and precipitation. Point shape indicates the classification of trait categories measured (e.g. morphological, physiological, performance/growth, or phenological) and size indicates the number of times that trait category was measured. The ellipsis highlights where the points are congregated and do not represent an analytical test.

Supplemental Tables

**SI Table 1.** Search terms used in the scholarly literature search for publications in EPIC.

| OR | ***AND*** | *OR* | ***AND*** | *OR* | ***NOT*** | *OR* | ***NOT*** | *OR* | ***NOT*** | *OR* | ***NOT*** | *OR* |
| --- | --- | --- | --- | --- | --- | --- | --- | --- | --- | --- | --- | --- |
| plant |  | seedling* |  | function* |  | pine |  | rice |  | rapeseed |  | South America* |
| plants |  | ontogen* |  | trait* |  | tree |  | faba bean |  | crop |  | Africa* |
|  |  | early-life |  | phenotyp* |  | ferns |  | chickpea |  | capsicum annuum |  | Europe* |
|  |  | early life |  | morpholog* |  | moss |  | mungbean |  | Alfalfa |  | Asia* |
|  |  |  |  | physiolog* |  | Arabidopsis |  | Oryza sativa |  | peanut |  | Australia* |
|  |  |  |  | phenolog* |  |  |  | wheat |  | barley |  | Canad* |
|  |  |  |  |  |  |  |  | Glycine max |  | passiflora edulis |  | Mexic* |
|  |  |  |  |  |  |  |  | Triticum |  | tomat* |  | Central America* |
|  |  |  |  |  |  |  |  | Cicer arietinum |  | Castor bean |  | China |
|  |  |  |  |  |  |  |  | *Maize* |  | Cabbage |  | Brazil |
|  |  |  |  |  |  |  |  | Chickpea |  | brassica oleracea |  | Spain |
|  |  |  |  |  |  |  |  | fibre flax |  | vigna radiata |  | Germany |
|  |  |  |  |  |  |  |  | lactuca sativa |  | vitis vinifera |  | Japan |
|  |  |  |  |  |  |  |  | Zea mays |  | wine |  | India |
|  |  |  |  |  |  |  |  | Rice |  | cocoa |  | France |
|  |  |  |  |  |  |  |  | sorghum bicolor |  | soybean |  | Italy |
|  |  |  |  |  |  |  |  | coffea arabica |  | Olea europaea |  | England |
|  |  |  |  |  |  |  |  | brassica napus |  | cucumber* |  | Iran |
|  |  |  |  |  |  |  |  | Sorghum |  | Beta vulgaris |  |  |
|  |  |  |  |  |  |  |  | lentil |  | beet |  |  |
|  |  |  |  |  |  |  |  | Cotton |  | Pisum sativum |  |  |
|  |  |  |  |  |  |  |  |  |  | pea |  |  |
|  |  |  |  |  |  |  |  |  |  | Brassica napa |  |  |
|  |  |  |  |  |  |  |  |  |  | Phaseolus vulgaris |  |  |
|  |  |  |  |  |  |  |  |  |  | bean |  |  |
|  |  |  |  |  |  |  |  |  |  | Lactuca sativa |  |  |
|  |  |  |  |  |  |  |  |  |  | lettuce |  |  |
|  |  |  |  |  |  |  |  |  |  | soybean |  |  |
|  |  |  |  |  |  |  |  |  |  | safflower |  |  |
|  |  |  |  |  |  |  |  |  |  | sorghum |  |  |
|  |  |  |  |  |  |  |  |  |  | alfalfa |  |  |
|  |  |  |  |  |  |  |  |  |  | Medicago sativa |  |  |

**SI Table 2.** Results from generalized linear models (GLMs) with a Poisson error distribution testing the number of studies measuring across years. Z and p-values are presented for each trait category (performance/growth, morphological, physiology, and phenological), trait type (above ground, below ground, and whole plant), and functional group (graminoids, forbs/herbs, shrubs).

| **Trait Category** | ***z*** | ***p-value*** |
| --- | --- | --- |
| performance/growth | -0.93 | >0.05 |
| morphological | 3.13 | >0.05 |
| physiology | -1.28 | >0.05 |
|  |  |  |
| **Trait Types** | ***z*** | ***p-value*** |
| above ground | 3.56 | >0.05 |
| below ground | 0.76 | >0.05 |
| whole plant | -0.99 | >0.05 |
|  |  |  |
| **Functional groups** | ***z*** | ***p-value*** |
| graminoids | 1.18 | >0.05 |
| forbs/herbs | 1.47 | >0.05 |
| shrubs | 0.20 | >0.05 |
